# Time-lapse digital cameras reveal contrasting environmental controls of leaf phenology in different caatinga physiognomies

**DOI:** 10.1007/s00484-026-03261-x

**Published:** 2026-07-13

**Authors:** Antonia Mirelle Lopes Marques, Desirée Marques Ramos, Leonor Patrícia Cerdeira Morellato, Kyle Graham Dexter, Bruna Alberton, Ítalo Antônio Cotta Coutinho

**Affiliations:** 1 Programa de Pós-Graduação em Sistemática, Uso e Conservação da Biodiversidade (PPGSis), Centro de Ciências, Departamento de Biologia, Universidade Federal do Ceará (UFC), Fortaleza, Brasil; 2 Center for Research on Biodiversity Dynamics and Climate Change, Department of Biodiversity, Institute of Biosciences, São Paulo State University (UNESP), Rio Claro, Brazil; 3 Instituto Tecnológico Vale, Belém, Pará Brasil; 4 Department of Life Sciences and Systems Biology, University of Torino, Turin, Italy; 5 School of GeoSciences, University of Edinburgh, Edinburgh, UK; 6 Royal Botanic Garden Edimburgo, Edimburgo, Reino Unido UK

**Keywords:** Carrasco, Crystalline caatinga, Climate variability, Phenocameras, Woody vegetation

## Abstract

Leaf phenology in Tropical Dry Forests (TDF) is strongly influenced by environmental factors; however, responses may vary according to edaphic, structural, and floristic differences. The Caatinga, one of the largest continuous TDF in the Americas, exhibits pronounced hydric seasonality and encompasses distinct physiognomies such as the carrasco (deciduous shrubland) and crystalline caatinga (xeric shrubland). Studies addressing phenological variation among these two vegetation types remain limited and largely based on direct human observation. Our study aimed to advance the understanding of phenological dynamics in Caatinga TDF by investigating the climatic factors influencing leaf production and fall in woody communities of carrasco and crystalline vegetations using repeated digital photographs. Daily monitoring (2020–2024) provided green chromatic coordinate time series. Climatic drivers of Caatinga phenology (photoperiod, precipitation, temperature, and atmospheric moisture) were analyzed using principal component analysis and temporally structured generalized additive models. Woody communities of crystalline caatinga and carrasco exhibited distinct phenological strategies reflecting different responses to climatic variability. Leaf phenology in both areas was mainly driven by precipitation but was also associated with temperature. Carrasco showed more stable and predictable dynamics, with lower interannual variation in start of growing season (SOS), end of growing season (EOS), and length of growing season (LOS), retaining leaves about 20 days longer and responding to relief of hydrothermal stress at the end of the dry season. Crystalline caatinga was highly sensitive to rainfall irregularity and precipitation pulses. These results suggest that crystalline caatinga is more vulnerable to increasing climatic variability, whereas carrasco appears more resilient.

**Supplementary Information:**

The online version contains supplementary material available at 10.1007/s00484-026-03261-x.

## Introduction

Leaf phenology is an essential component of community functioning, as it regulates key processes such as carbon exchange, water and energy fluxes, and nutrient cycling, which together affect community productivity and climatic balance (Reich 1995; Polgar and Primack 2011; Richardson et al. 2013). Therefore, understanding leaf phenology is essential for predicting how ecosystems will respond to increased climate variability (Forrest and Miller-Rushing 2010; Alberton et al. 2019) as well as to global environmental changes (Morellato et al. 2016; Piao et al. 2019).

In Tropical Dry Forests (TDFs), leaf phenology is of utmost importance as strong climatic seasonality imposes plants severe constraints on their physiological activities (Borchert 1994; Chaturvedi et al. 2021). TDFs occur across multiple continents and are among the tropical ecosystems most sensitive to changes in rainfall regimes and temperature (Allen et al. 2017; Siyum 2020; Ferreira et al. 2024). In these environments, leaf flushing and senescence are mainly related to water availability, photoperiod, and thermal conditions, limiting the duration of growth and photosynthetic activity to the short rainfall period (Reich 1995; Alberton et al. 2019, 2023; Lima et al. 2021). However, even constrained by strong water seasonality, TDFs exhibit a diversity of phenological patterns as leaf flushing and senescence may also vary under the influence of other factors, such as soil properties, root depth (Borchert 1994; Wright 1996; Neves et al. 2017) and species functional traits (Borchert 1994; Chaturvedi et al. 2021; Lima et al. 2021). For instance, in areas with shallow soils there is a rapid tendency for water loss, restricting leaf production to the rainy season (Medeiros et al. 2022). However, evapotranspiration and air temperature may also influence leaf phenological patterns, although their effects are less evident (Vico et al. 2015; Medeiros et al. 2022; Alberton et al. 2023).

The Caatinga is considered one of the largest continuous TDFs in the Americas (Murphy and Lugo 1986; Queiroz et al. 2017; Fernandes et al. 2020; Moro et al. 2024), characterized by intense solar radiation, high evapotranspiration, high temperatures, and water deficit, associated with annual precipitation that generally does not exceed 1,000 mm and that has become increasingly erratic (Prado 2003; Moro et al. 2016, 2024; Queiroz et al. 2017; Moura et al. 2020). In addition, the Caatinga is facing increasing local anthropogenic pressures and is severely affected by climatic changes and desertification (Alves et al. 2008; Silva et al. 2019). Due to its large spatial extent, environmental heterogeneity, and pronounced climatic constraints, the Caatinga represents a natural laboratory for investigating phenological responses in TDF (Fernandes et al. 2022; Ferreira et al. 2024; Franca Rocha et al. 2024).

Besides climate, Caatinga vegetation is also influenced by edaphic factors (Dexter et al. 2018; Moro et al. 2024; Brunello et al. 2025), encompassing crystalline basins typically with shallow, stony soils and sedimentary basins with deeper, sandier soils, leading to the formation of distinct physiognomies with differences in structure, functioning, and floristic composition (Moro et al. 2015, 2016, 2024; Dexter et al. 2018; Wells et al. 2025). Among the predominant physiognomies are the Caatinga sensu stricto, or crystalline caatinga, which is associated with the soils of the Sertaneja Depression (Moro et al. 2016; Queiroz et al. 2017), and the Sedimentary Caatinga, also known as carrasco (Araújo and Martins 1999; Moro et al. 2016). Crystalline caatinga occurs predominantly in lowlands (200–600 m a.s.l.) presenting shallow, fertile, and stony soils, where deciduous and thorny vegetation, with a high representation of families such as Fabaceae, Euphorbiaceae and Cactaceae (Rodal et al. 2008; Moro et al. 2014, 2024; Queiroz et al. 2017; Fernandes et al. 2020). In contrast, the carrasco occurs at higher altitudes (700–900 m a.s.l.) with deep, sandy, oligotrophic soils and is characterized by dense, predominantly non-thorny shrubby vegetation with a high richness of woody species, especially within the Fabaceae, Euphorbiaceae and Myrtaceae (Araújo and Martins 1999; Vasconcelos et al. 2010; Araújo et al. 2011; Queiroz et al. 2017). These two physiognomies have been the subject of several studies (Araújo et al. 2005, 2007, 2011; Santos et al. 2011; Moro et al. 2014, 2015, 2024). However, such studies focused mainly on understanding their floristic composition, structure, and soil–vegetation relationships. For carrasco vegetation specifically, we found only one study describing phenological patterns based on direct human observations (Vasconcelos et al. 2010).

Even though leaf phenological studies are found in the literature for the crystalline caatinga and carrasco, most studies are based on direct human observations, which are subjective and therefore limited on accuracy and overall spatial coverage (Alberton et al. 2017; Liu et al. 2021). These limitations were recently overcome by the use of time-lapse digital photographs, called phenocameras, an effective alternative for long-term leaf phenological monitoring (Alberton et al. 2017; Richardson et al. 2018; Morellato et al. 2024). This fine-scale, near-surface monitoring is particularly valuable in seasonal tropical vegetation, where leaf phenological transitions can occur within a few days, and leaf flushing may respond to rainfall pulses within 7 days (Alberton et al. 2019, 2023), limiting detection by coarse temporal-resolution approaches. High-frequency observations are therefore essential to accurately capture rapid canopy dynamics and to better understand how environmental drivers such as precipitation, photoperiod, and temperature regulate leaf flushing, senescence, and overall canopy seasonality (Camargo et al. 2018; Alberton et al. 2019; Medeiros et al. 2022; Ramos et al. 2023).

In context, the present study aims to advance the understanding of phenological dynamics of woody vegetation in two distinct Caatinga physiognomies, crystalline caatinga and carrasco, using a four-year phenocamera time series. Specifically, we aim to: (i) compare the timing of leaf flushing, senescence, and the length of the growing season between crystalline and carrasco; and (ii) identify the environmental factors - photoperiod, precipitation, temperature, and atmospheric water demand (potential water deficit and vapor pressure deficit) - shaping phenological patterns in tree communities of both physiognomies.

We hypothesize that carrasco, due to its deeper and sandier soils, exhibits a longer leaf growing season than crystalline Caatinga. We further expect leaf phenology in carrasco to be less dependent on initial precipitation pulses and more strongly influenced by photoperiod and evapotranspiration. In contrast, crystalline caatinga, with its shallow and stony soils, is predicted to show phenological patterns more tightly synchronized with precipitation and air temperature, with rapid leaf flushing following the first rainfall pulses and accelerated leaf senescence after the rainy season. By comparing these contrasting Caatinga physiognomies occurring under similar climatic conditions, this study provides a framework for understanding phenological controls in TDF.

## Materials and methods

### General characterization of the study area

**Fig. 1:**
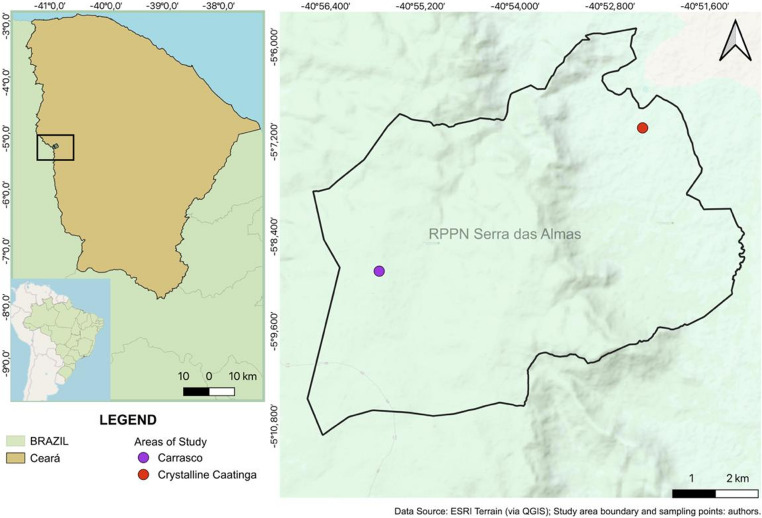
Study area at the Reserva Particular do Patrimônio Natural Serra das Almas (RPPN Serra das Almas), Ceará State, northeastern Brazil, highlighting the phenocamera sampling points in two caatinga physiognomies, carrasco and crystalline caatinga

The study was conducted in a Caatinga area of the Brazilian semiarid region, within the Serra das Almas Natural Reserve (RNSA, Fig. 1), located in the state of Ceará, in the central-southern region of the Ibiapaba Plateau. Leaf phenology of the native woody vegetation was monitored from 2020 to 2024 in two Caatinga physiognomies: crystalline (5°07′01.5″ S; 40°52′23.2″ W) and carrasco (5°08′47.5″ S; 40°55′41.3″ W). The areas are located approximately 7 km apart. Climatic data for each site during the period corresponding to phenological monitoring was obtained from the Climate Engine platform (ClimateEngine.org), derived from the ERA5 reanalysis (Land – 11.1 km – Daily, European Centre for Medium-Range Weather Forecasts – ECMWF). The variables obtained from the platform included mean (Tmean), minimum (Tmin), and maximum (Tmax) air temperature (°C), mean dew point temperature (°C), and potential water deficit (PWD; Hargreaves/PET, mm). Total daily precipitation (rainfall, mm) was collected locally using a Ville de Paris rain gauge (IPL002).

For floristic characterization, a census was conducted in 2022 within 0.5 ha plots established in each physiognomy (crystalline caatinga and carrasco), totaling 1 ha of sampled vegetation. All woody individuals with diameter at ground level (DGL) ≥ 5 cm were sampled and identified to species level. Data collection followed the protocols described in the Field Manual for Plot Establishment and Remeasurement developed by the DRYFLOR network (Moonlight et al. 2021; DryFlor et al. 2022). A phytosociological analysis was carried out to obtain the importance value index (IVI) (Curtis and McIntosh 1951) of the 80% most representative species. The selection of species representing 80% of cumulative IVI aimed to focus on the dominant species responsible for the main structure of the plant community. Moreover, in both studied physiognomies, the phenocameras were directed towards the species comprising most of this 80%.

#### Carrasco

**Fig. 2:**
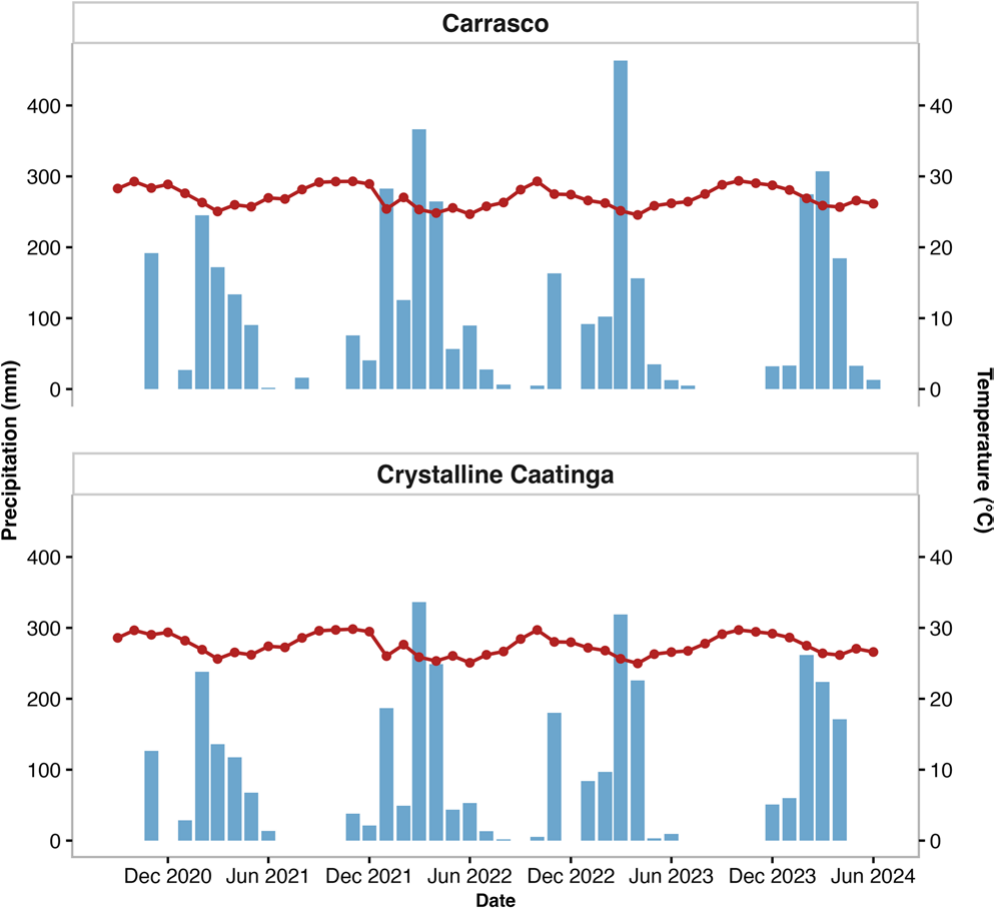
Monthly average temperature (red lines) and total rainfall (bars) for each study site: carrasco and crystalline caatinga (2020–2024)

The carrasco occurs at an altitude of approximately 750 m above the sea level, under a mild hot semi-arid climate (As) (Alvares et al. 2013), with deep Quartzarenic Neosols that are sandy-loam, nutrient-poor, and characterized by a high proportion of sand and low clay and silt contents (Araújo Filho et al. 2022; Marques 2024; Brunello et al. 2025). Monthly precipitation ranged from 0 to 460 mm. During the rainy season, usually occurring from November to May, rainfall generally reached 100–300 mm, whereas during the dry season, usually occurring from June to October, values remained below 30 mm. Mean air temperature ranged from approximately 24.5 to 29.5 °C. (Fig. 2). It is important to notice that during the rainy season dry months could occur as the rains were erratic even during the rainy season. In carrasco vegetation, *Eugenia nordestin*a L.R.V. Santos & I.R. Costa presented the highest IVI (90.77), followed by *Pityrocarpa moniliformis* (Benth.) Luckow & R.W.Jobson (46.34), *Hymenaea velutina* Ducke (25.67), *Agonandra brasiliensis* Miers ex Benth. & Hook.f. (16.7), *Senegalia polyphylla* (DC.) Britton & Rose (13.72), *Campomanesia* sp. Ruiz et Pav. (13.69), *Eugenia stictopetala* Mart. ex DC. (11.94), *Guapira graciliflora* (Mart. ex J.A.Schmidt) Lundell (10.97), *Hymenaea eriogyne* Benth. (8.37), and *Mimosa verrucosa* Benth. (8.26).

#### Crystalline caatinga

The crystalline caatinga occurs at an altitude of approximately 300 m above the sea level, under a hot semiarid climate (BSh) (Alvares et al. 2013), and soils classified as Chromic Luvisols (Brunello et al. 2025). These soils range from shallow to moderately deep, are clayey, but have low water-holding capacity, high susceptibility to erosion, and surface stoniness (Santos et al. 2018; Araújo Filho et al. 2022; Brunello 2022). Monthly precipitation reached up to 330 mm, with the largest rainfall pulses concentrated in the first months of the year. During the rainy season, usually occurring from November to April, precipitation ranged from 80 to 250 mm, whereas during the dry season, usually occurring from May to October, values remained below 10 mm. As for the carrasco, irregular rainfall occurred in the crystalline caatinga during the rainy season, allowing dry months even within such a season. Mean air temperature showed low seasonal variation, ranging from approximately 25 to 30 °C (Fig. 2). In crystalline caatinga, *Cordia* sp. L. showed the highest Importance Value Index (IVI = 138.63), followed by *Mimosa caesalpiniifolia* Benth. (49.4), *Bauhinia cheilantha* (Bong.) Steud. (24.7), *Piptadenia retusa* (Jacq.) P.G.Ribeiro, Seigler & Ebinger (22.2), and *Croton blanchetianus* Baill. (16.7).

### Climate data

Climatic data for each site was obtained as described in Sect.  2.1. Daily thermal amplitude (Tamp) was calculated using Tmax and Tmin data, and vapor pressure deficit (VPD, kPa) was estimated as the difference between saturation vapor pressure (calculated based on Tmean) and actual vapor pressure (derived from mean dew point temperature) (Tian et al. 2024). Total daily precipitation (rainfall, mm) was collected locally using a Ville de Paris rain gauge (IPL002). The effects of precipitation were tested using lags of cumulative precipitation over the previous seven days (Rainfall_lag7) and 30 days (Rainfall_cum30), following Alberton et al. (2019), who demonstrated the importance of these hydrological variables in driving leaf phenological transitions in the Caatinga. Day length was estimated as the number of daylight hours and calculated using the geosphere package in R (v. 1.5–10) for the latitude of each site. The precipitation accumulation metrics used to compare with the transition dates followed the approach proposed by Godlee et al. (2024).

### Camera setup and image processing

The phenological monitoring was conducted from 2020 to 2024. In 2020, two phenocameras (Bushnell, model 119836 C) were used for phenological monitoring in the crystalline caatinga and Carrasco, with one camera in each location, installed on trees at 6 m and 10 m high, respectively, capturing at least 40% (2,000 m^2^) and 50% (5,000 m^2^) of the entire plot. Due to malfunctions in the original units. in January 2023, the camera in the carrasco was replaced with a Bushnell 119,949 C, and in february 2024, the camera in the crystalline caatinga was replaced with a Bushnell 119,936 C. Images (JPEG, 1280 × 960 pixels) were acquired daily over four growing seasons, from 2020 to 2024, with three images per hour recorded between 06:00 and 18:00 (UTC − 3; Coordinated Universal Time). Images with partial or total obstructions in the camera field of view were removed. Regions of interest (ROIs) were defined to extract vegetation indices, encompassing the woody vegetation stratum of the community, including all plants within the camera field of view.

### Data analysis

#### Camera-based remote phenological index

The Green Chromatic Coordinate index (Gcc) was extracted for each ROI based on the color channels of the digital images: red, green, and blue (RGB (Woebbecke et al. 1995). This index was adopted due to its reliability in capturing changes in leaf flushing and senescence while reducing variations in illumination caused by solar angle and camera settings (Gillespie et al. 1987; Richardson et al. 2009; Migliavacca et al. 2011; Alberton et al. 2014). To avoid distortions in green values, the *per90* method was applied, considering images acquired between 10:00 and 14:00 h (UTC − 3; Coordinated Universal Time) (adapted from Sonnentag et al. 2012; according to Ramos et al. (2023). Due to camera replacement, multiple cameras monitored the same field of view, and their GCC time series were merged. Before merging, values were standardized (mean = 0, variance = 1) to ensure consistency, as the cameras were not calibrated.

#### Phenological metrics

The Gcc time series was used to calculate phenological transition dates (PTD), including the start of the growing season (SOS), the end of the growing season (EOS), and the length of the growing season (LOS) (Alberton et al. 2014, 2019; Richardson 2019). A generalized additive model (GAMs) was fitted, modeling Gcc as a function of time (days), the later used as a smooth variable, allowing the construction of phenological curves (Klosterman et al. 2014; Filippa et al. 2016; Camargo et al. 2018; Alberton et al. 2019). To account for temporal autocorrelation in the residuals, we included an autoregressive moving average (ARMA) structure for the daily observations, using two consecutive days (*p* = 2). From these fitted curves, significant points of variation were identified through derivative analysis. A total of 10,000 simulations were used to estimate 95% confidence intervals and to determine when the derivatives were statistically different from zero (Alberton et al. 2019).

Based on the established confidence intervals, periods of Gcc increase and decrease were delineated. SOS was defined as the point at which Gcc reached approximately 50% of the total increase in greenness along the phenological curve, indicating the onset of leaf flushing. EOS corresponded to the point at which approximately 80% of the decrease in Gcc was observed, characterizing leaf senescence (Alberton et al. 2019; Ramos et al. 2023). LOS was calculated as the interval, in days, between SOS and EOS. These results were complemented by a visual inspection of the images, associating increases in greenness with leaf flushing and decreases with leaf senescence, to ensure the reliability of SOS, EOS, and LOS estimates. All analyses were performed using the R software environment (R Core Team 2022).

The calculation of SOS, EOS, and LOS followed Eqs. (1)–(5). Periods of significant Gcc increase and decrease were identified from the derivatives of the phenological curve, defining $\:SO{S}_{start}\:$ (increase) and $\:SO{S}_{end\:}$(decrease). $\:{D}_{SOS}\:$ and $\:{D}_{SOS}\:$ represent the duration (in days), of the increasing and decreasing periods, respectively. $\:SOS$ was defined as the day corresponding to 50% of the total Gcc increase, and the *EOS* as the day corresponding to 80% of the total Gcc decrease. $\:LOS$ was calculated as the interval (in days) between SOS and EOS, and T corresponds to the total number of days in the year, fixed at 365.

1 $$\:{\boldsymbol{D}}_{\boldsymbol{S}\boldsymbol{O}\boldsymbol{S}}=\:\left(\boldsymbol{T}\:-\:\boldsymbol{S}\boldsymbol{O}{\boldsymbol{S}}_{\boldsymbol{s}\boldsymbol{t}\boldsymbol{a}\boldsymbol{r}\boldsymbol{t}}\right)+\:\boldsymbol{S}\boldsymbol{O}{\boldsymbol{S}}_{\boldsymbol{e}\boldsymbol{n}\boldsymbol{d}}$$

2 $$\:SOS\:=\:SO{S}_{start}+\:0.5\:\times\:\:{D}_{SOS}$$

3 $$\:{D}_{EOS}=\:\left(T\:-\:EO{S}_{start}\right)+\:EO{S}_{end}$$

4 $$\:EOS\:=\:EO{S}_{start}+\:0.8\:\times\:\:{D}_{EOS}$$

5 $$\:LOS\:=\:EOS\:-\:SOS$$

#### Statistical analysis

Using the phenological metrics extracted from the Gcc time series, we tested which environmental variables influenced vegetation phenological dynamics for each study site separately. To this end, we fitted generalized additive models (GAMs) (Wood 2017), considering Gcc as the response variable and environmental variables as predictors. Day of year (DOY) was included as a cyclic smooth term (bs = “cc”) to model annual seasonal patterns, in addition to a smooth term to represent long-term temporal trends (t).

Due to the temporal structure of the data, the presence of autocorrelation was assessed, and GAMs were fitted with a first-order autoregressive correlation structure (AR1) using the gamm function from the *mgcv package*, with parameter estimation performed by restricted maximum likelihood (REML) (Wood 2011, 2017). This approach improves model stability and reduces the tendency towards overfitting (Wood 2017; Pedersen et al. 2019). For each study area, different models were tested using combinations of daily climatic variables, as well as alternative models with aggregated climatic variables. All models were subjected to a series of diagnostic checks to evaluate their adequacy. Model selection was based on the Akaike Information Criterion (AIC), with the model presenting the lowest AIC considered the best-fitting model (Akaike 1974).

Covariates were modeled using penalized regression splines, with different numbers of basic functions (k) defined according to the expected variability of each variable. To assess whether the chosen basis dimensions were sufficient, we used the gam.check function, examining the k-index and the effective degrees of freedom (EDF) (Wood 2017). Residual structure was inspected using the autocorrelation function (ACF). To avoid redundancy among smooth terms, concurvity was evaluated for each model using the concurvity function. To reduce collinearity among highly correlated climatic variables, a principal component analysis (PCA) was applied separately for each study area (Dormann et al. 2013), considering the climatic variables Rainfall_lag7, Rainfall_cum30, Tmean, VPD, Tamp, PWD, and photoperiod. The resulting principal components were incorporated as smoothed covariates in the GAMs, representing integrated climatic gradients.

After comparing models using raw climatic variables with models using principal components, those including PC1 and PC2 for carrasco and PC1 and PC3 for crystalline Caatinga showed better performance and lower AIC values. Subsequently, the loadings of each principal component were examined to identify the variables with the greatest contribution and statistical significance within each PCA. To facilitate the ecological interpretation of the nonlinear relationships between Gcc and each covariate, partial effects of the smooth terms were generated and visualized individually for each study area. All analyses were performed in the R software environment(R Core Team 2022) using the packages *mgcv* (Wood 2017), *gratia* (Simpson 2019), *nlme*(Pinheiro and Bates 1999) and *tidyverse* (Wickham et al. 2019).

## Results

### Detection of growing seasons

**Fig. 3:**
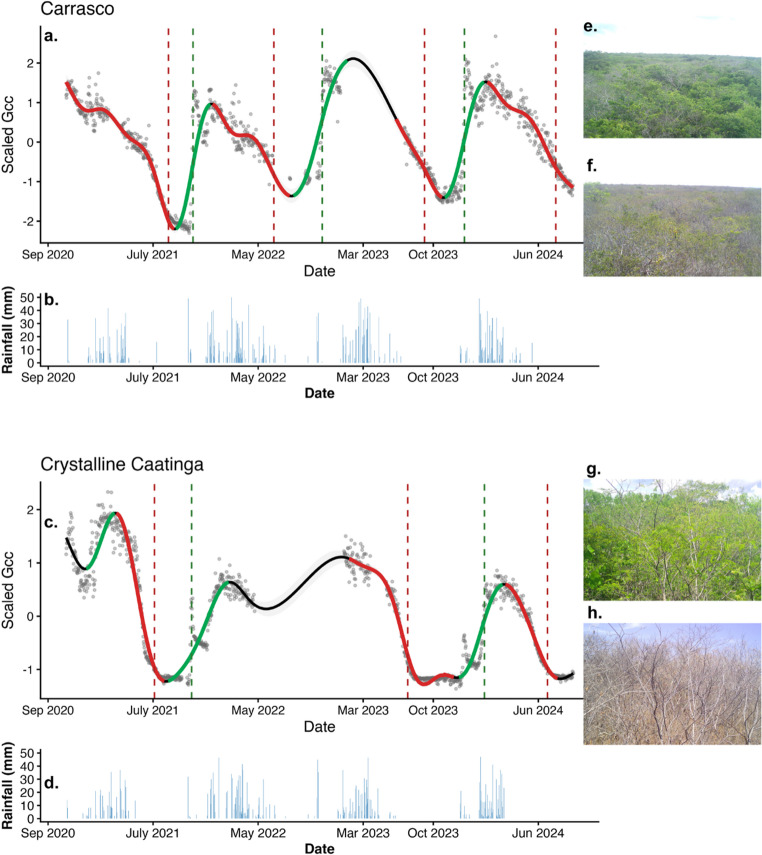
Leaf phenological dynamics and precipitation in carrasco and crystalline caatinga (2020–2024). (a, c) GAM-Fitted models of Gcc over time (black lines) showing the periods of derivative increase (green), and decrease (red), and the start (SOS) and end (EOS) of the growing season (dashed lines). The grey dots represent the observed data. (b, d) Accumulated daily precipitation (mm). (e–h) Phenocamera images of carrasco and crystalline caatinga during rainy (e, g,) DOY 56/2024 and dry seasons (f, g,) DOY 310/2024

Carrasco and crystalline caatinga exhibited different seasonal patterns of leaf flushing, leaf fall, and growing season duration during the rainy season across the analyzed years (Fig. 3), showing contrasting responses to interannual variability in water dynamics. Accumulated precipitation during the rainy seasons was consistently higher in the carrasco than in the crystalline caatinga across the monitored years (2021–2024). In carrasco, accumulated rainfall ranged from 661.2 to 1301.8 mm, whereas in the crystalline caatinga it varied from 570.6 to 919 mm. The largest difference between vegetation types occurred during the 2022 rainy season, when precipitation in carrasco exceeded that of the crystalline caatinga by 382.8 mm. In the other years, the differences were smaller but still consistent, ranging from 69.1 to 90.6 mm.

In carrasco, the leaf flushing was more consistent among years, generally occurring at the end of the dry season and coinciding with the onset of rainfall, always following the first precipitation pulses. For instance, in 2021, SOS occurred on 14 November (DOY 318) and, in 2022, on 18 November (DOY 322) (Fig. 3). In 2023, SOS occurred on 29 December (DOY 363), representing a delay of approximately 45 days compared to previous years (Fig. 3). Contrastingly, in crystalline caatinga, the leaf flushing pattern showed greater variability. In 2021, SOS occurred earlier, on 10 November (DOY 314), whereas during the 2023–2024 cycle this pattern was disrupted, resulting in a delay of 69 days, with SOS occurring only on 24 February 2024 (DOY 55).

The leaf fall was less synchronous than leaf flushing. Although it occurred after the end of the rainy season at both sites, leaves in carrasco were retained for approximately 20 days longer than in crystalline caatinga. In carrasco, EOS showed greater interannual variability, occurring on 5 September 2021, 3 July 2022, 6 September 2023, and 16 September 2024, spanning a range between DOY 184 and 259. In crystalline caatinga, EOS varied less among years, being concentrated between DOY 201 and 235. In 2021, EOS occurred on 27 July, in 2023 on 20 July, and in 2024 on 23 August, indicating earlier leaf senescence relative to carrasco.

Because of the contrasting SOS and EOS patterns, the length of the growing season (LOS) differed markedly between the study areas. In carrasco, LOS was 231 days in the 2021–2022 cycle, 292 days in 2022–2023, and 261 days in 2023–2024, average LOS in the carrasco considering the three years was ~ 261 days (± 30,5 days), reflecting a variable leafing window across years. In crystalline caatinga, LOS could be estimated only for the 2023–2024 cycle and was 180 days, 80 days shorter than in carrasco.

### Environmental drivers of community leaf phenology

**Fig. 4:**
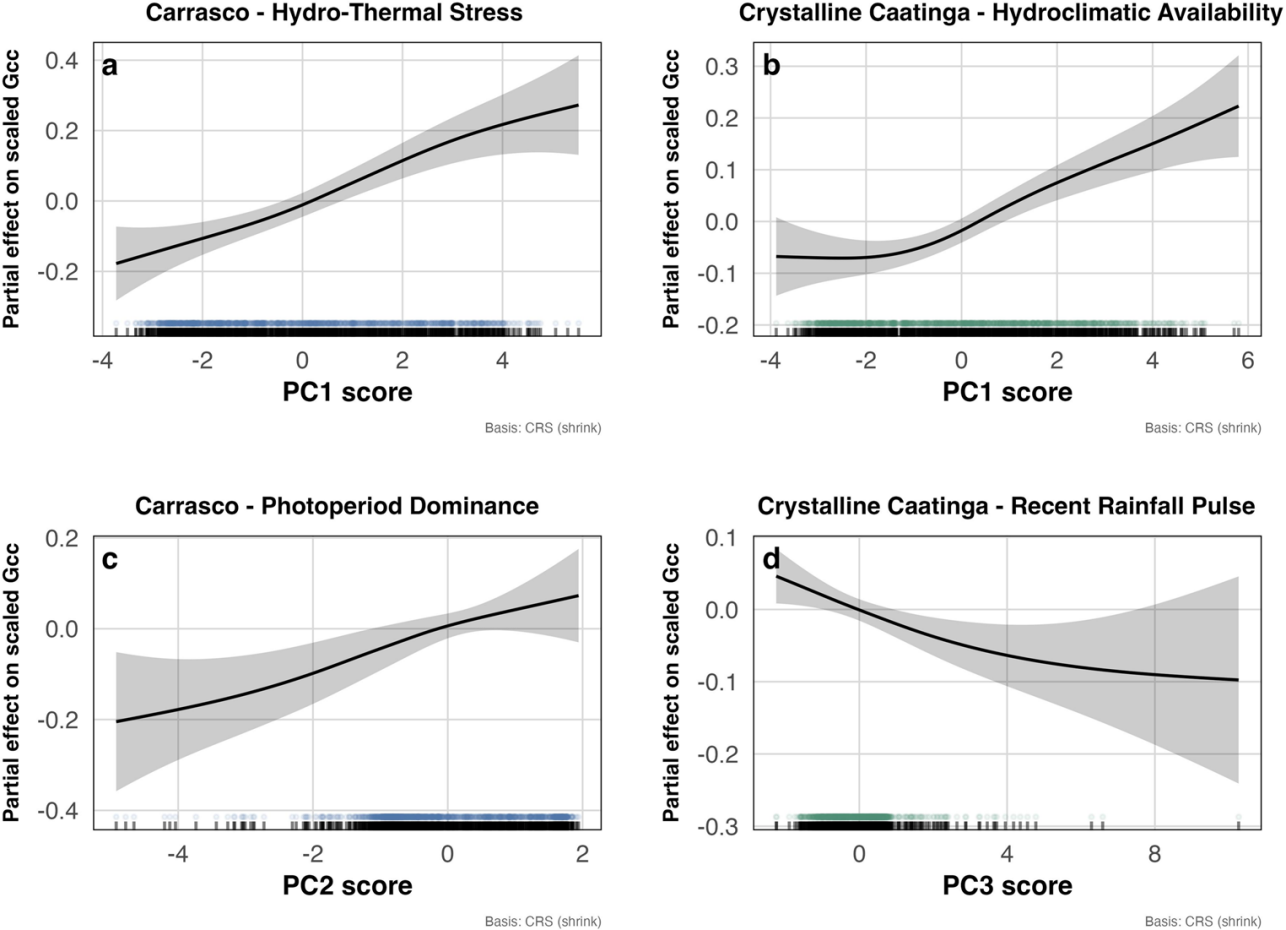
Partial effects of principal components on standardized Gcc (scaled_Gcc) estimated by GAMs for carrasco and crystalline caatinga. Panels show (**a**) the hydrothermal stress effect in carrasco, (**b**) the hydroclimatic availability effect in crystalline caatinga, (**c**) the photoperiod dominance effect in carrasco, and (**d**) the recent precipitation pulse effect in crystalline Caatinga. Black lines represent smoothed partial effects, gray bands indicate 95% confidence intervals, and rug marks show the distribution of observed values along the principal component axes

In both areas, the first principal component (PC1) represented the dominant climatic gradient, explaining the largest proportion of total variance (Table 1). In carrasco, PC1 showed strong negative loadings for Tmean, VPD, Tamp, and PWD (Fig. S1; Table S1) and was interpreted as a hydrothermal stress gradient, defined as a gradient primarily driven by high temperatures and drier atmospheric air (high evaporative demand). The partial effect curve showed a monotonic increasing pattern, indicating that a gradual reduction in aridity and evapotranspiration was associated with increased leaf production (Fig. 4a). Accordingly, negative PC1 values characterized periods of higher temperatures, drier air, and greater water deficit, whereas increasing PC1 values were associated with higher Gcc. This relationship was positive and statistically significant (Table 2), and the model showed high explanatory power (R² = 0.951).

**Table 1:** PCA summary for carrasco and crystalline caatinga, showing standard deviation and explained and cumulative variance of components used in the models

Area	PC	SD	Explained variance (%)	Cumulative variance (%)
Carrasco	Hydrothermal stress gradient	2.065	60.93	60.93
	Seasonal photoperiod gradient	1.101	17.31	78.23
Crystalline caatinga	Hydroclimatic availability gradient	2.042	59.55	59.55
	Recent rainfall pulse gradient	0.907	11.74	71.29

*SD* Standard Deviation

**Table 2:** Summary of GAMs for Gcc dynamics in carrasco and crystalline caatinga, showing smooth terms included in each model, effective degrees of freedom (EDF), F values, and significance levels. Adjusted R² indicates overall model performance. Models accounted for temporal structure, annual seasonality, and selected climatic principal components for each physiognomy. Significance levels: *** *p* < 0.001

Area	Term	EDF	F	Significance	Adjusted *R*²
Carrasco	s(t)	47.461	61.510	***	**0.951**
	s(doy)	16.025	2.160	***	
	s(PC1)	2.637	0.712	***	
	s(PC2)	1.610	0.161	***	
Crystalline caatinga	s(t)	51.106	83.844	***	**0.979**
	s(doy)	9.745	0.529	***	
	s(PC1)	3.264	1.378	***	
	s(PC3)	1.382	0.351	***	

Crystalline caatinga PC1 showed a value range similar to that in carrasco but primarily represented a hydroclimatic availability gradient, defined as a gradient integrating both atmospheric conditions and precipitation-related variables that reflect water availability. The smoothed curve revealed a nonlinear response (Fig. 4b), with low Gcc under drier conditions (negative values) and a rapid increase in leaf production once water availability increased, remaining high under wetter conditions (positive values). This effect was highly significant (Table 2), indicating that Gcc responded more strongly to water availability than to immediate reductions in atmospheric stress. Overall, PC1 represented the main climatic gradient in both areas, reflecting reduced aridity and hydrothermal stress in carrasco and increased water availability in crystalline caatinga.

The remaining components showed contrasting effects between areas, with PC2 being significant in carrasco and PC3 in crystalline caatinga. In carrasco, PC2 was strongly associated with photoperiod, with a secondary contribution from precipitation (Fig. S1; Table S1), and was defined as a seasonal photoperiod gradient. The partial effect curve showed a positive but low-intensity effect on Gcc (Fig. 4c), indicating that seasonal light variation modulated leaf production, although with weaker influence than the main climatic gradient (PC1). In contrast, PC3 in crystalline caatinga was related to recent precipitation and showed a decreasing effect on Gcc (Fig. 4d), being defined as a recent rainfall pulse gradient. Gcc values were only maintained under sustained rainfall conditions, declining as precipitation decreased or became more erratic, suggesting that leaf phenology in crystalline caatinga was highly sensitive to short-term rainfall fluctuations, particularly when pulses were not sustained over time.

**Fig. 5:**
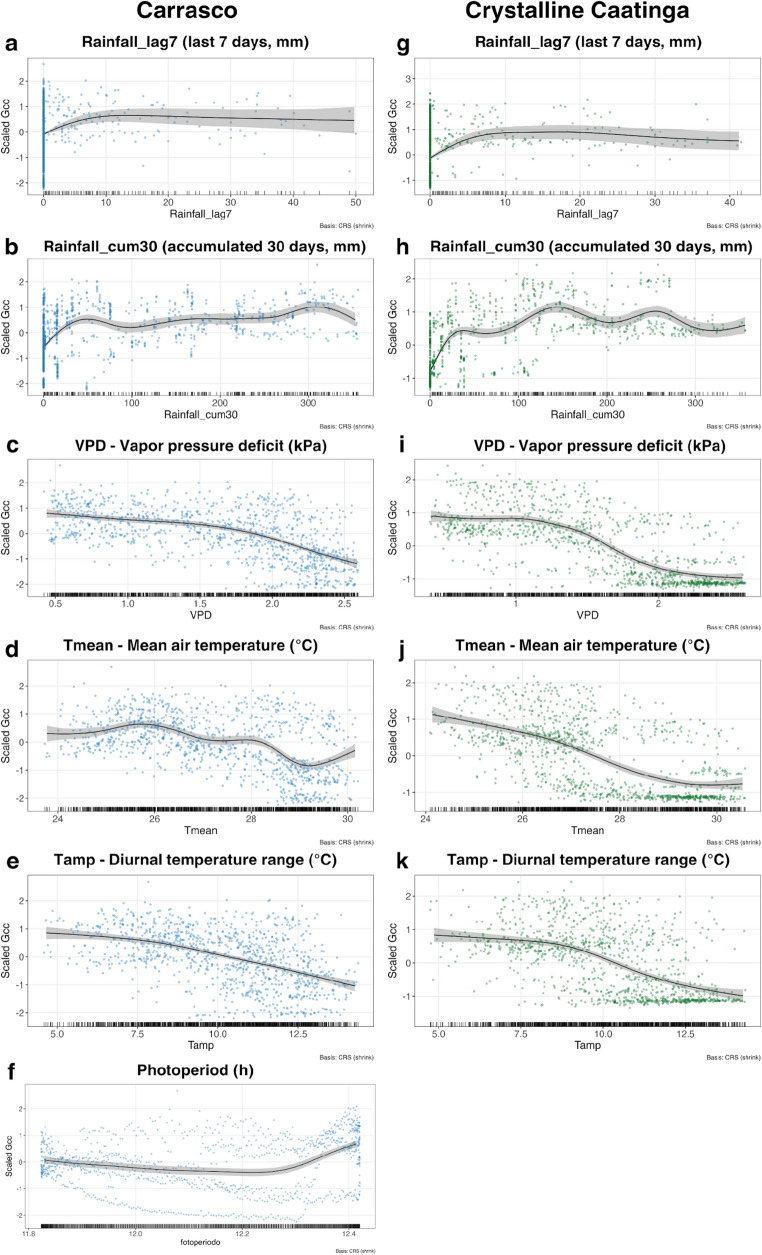
Partial effects of climatic variables on scaled_Gcc estimated by GAMs for carrasco (**a–f**) and crystalline (**g–k**) caatinga. Panels show effects of Rainfall_lag7, Rainfall_cum30, vapor pressure deficit (VPD), mean air temperature (Tmean), daily thermal amplitude (Tamp), and photoperiod. Black lines represent smoothed partial effects with 95% confidence intervals (gray), points indicate observations, and rug marks show data distribution along environmental gradients

To complement the interpretation of the principal components, we evaluated the individual effects of each climatic variable on Gcc at both sites (Fig. 5). In carrasco, responses followed a more gradual pattern. Recent precipitation (Rainfall_lag7) showed a positive effect on Gcc, with increases at values between 10 and 20 mm, followed by stabilization at higher values (Fig. 5a). Thirty-day accumulated precipitation was also significant and contributed to Gcc stability at values above 50 mm (Fig. 5b). Leaf production was concentrated at mean air temperatures (Tmean) between 24 and 26 °C, whereas higher temperatures were associated with reduced Gcc (Fig. 5d). High values of vapor pressure deficit (VPD) and thermal amplitude (Tamp) showed negative effects on Gcc, confirming reduced leaf production under drier conditions and greater temperature variability (Fig. 5c, e). Photoperiod showed a weaker positive effect, acting as a secondary modulator, with Gcc increasing when day length exceeded 12.4 h and decreasing under shorter days (Fig. 5f).

In crystalline caatinga, values of recent precipitation (Rainfall_lag7) were also concentrated around 10–20 mm. However, the response curve was slightly steeper than in carrasco, suggesting greater sensitivity of leaf production to recent rainfall pulses (Fig. 4g). The response to 30-day accumulated precipitation showed lower stability, with more pronounced oscillations than in carrasco (Fig. 4h). Mean air temperatures above 27 °C (Fig. 3j), VPD values exceeding ~ 1.5 kPa (Fig. 4i), and higher thermal amplitude (Tamp; Fig. 4k) showed decreasing effects on Gcc, with a more abrupt decline in leaf phenology compared to carrasco.

## Discussion

Our results revealed marked contrasts in leaf phenological dynamics between carrasco and crystalline caatinga, expressed in the timing of leaf flushing and senescence, the length of the growing season, and the sensitivity to interannual climatic variability. Carrasco exhibited a more stable onset of leaf flushing and a longer growing season, whereas crystalline caatinga showed greater variability, shorter growing seasons, and earlier senescence. Such differences were consistently reflected in the climatic controls identified by the GAM analyses, with hydrothermal stress and photoperiod modulating leaf production in carrasco, and water availability and short-term rainfall pulses exerting stronger control in crystalline caatinga. Overall, these results indicate that crystalline caatinga is more sensitive than carrasco to temperature variation and increased evapotranspiration, requiring more continuous rainfall to sustain leaf production and maintenance.

### Leaf phenological patterns of the communities

The woody communities of carrasco and crystalline caatinga exhibited interannual variation in seasonal patterns of leaf flushing, senescence, and leaf duration, reflecting the responses of these physiognomies to interannual hydrological variability. Such patterns are widely reported for seasonal ecosystems (Pezzini et al. 2014; Alberton et al. 2019; Paloschi et al. 2020; Ramos et al. 2023). In seasonally dry ecosystems, leaf production is generally associated with water availability, with plants responding rapidly to initial rainfall pulses after periods of severe water stress (Reich and Borchert 1984; Borchert 1994; Machado et al. 1997; Alberton et al. 2019). However, some species may exhibit phenological patterns driven not only by precipitation but also by other climatic cues, resulting in different degrees of predictability and interannual variability (Kushwaha et al. 2011).

Carrasco exhibited a more predictable seasonal dynamic, reduced dependence on immediate rainfall events, lower interannual variation in SOS, EOS, and LOS than crystalline caatinga. Synchronous leaf pattern has been previously reported for carrasco (Vasconcelos et al. 2010), where species tend to produce leaves at the end of the dry season and the onset of the rainy season. Our four-year long study strengthens previous findings (Vasconcelos et al. 2010), based on a single year. In TDFs, such predictable phenological patterns are linked to greater tolerance to water stress and likely represent a conservative water-use strategy (Sterck et al. 2011; Hallmark et al. 2024).

In contrast, crystalline caatinga phenology appeared to be more sensitive than carrasco to changes in rainfall regime, particularly in years with delayed precipitation. This pattern is consistent with those reported for other Caatinga/Brazilian dry forests communities under stronger water limitation (Pezzini et al. 2014; Medeiros et al. 2022; Ramos et al. 2023), that tend to respond faster to rainfall irregularity. Such responses suggest an opportunistic use of short periods of water availability, with vegetative activity occurring rapidly following rainfall events (Vico et al. 2015; Feldman et al. 2021). Overall, in TDFs, the onset of leaf production and other phenophases has been strongly responsive to water availability and its temporal distribution (Williams et al. 2008; Kushwaha et al. 2011; Morellato et al. 2024; Santos et al. 2025).

This contrasting phenological pattern between the two studied areas is most likely influenced by edaphic characteristics. As shallow Chromic Luvisols soils (~ 1 m deep) occur in the crystalline caatinga whereas deeper Quartzarenic Neosols soils in the carrasco (~ 2 m deep) (Brunello 2022), this difference likely reduces the availability of soil water reserves in the crystalline caatinga compared to the carrasco (Santos et al. 2014; Wright et al. 2021). Although both areas experience similar seasonal rainfall patterns, the carrasco tends to receive slightly greater accumulated precipitation (69.1–90.6 mm) and shows higher interannual variability in rainfall totals (exceptionally reaching 382.8 mm in one year). In addition to occurring on shallower soils, the crystalline caatinga is characterized by clayey soils with high surface stoniness and greater susceptibility to erosion compared to the carrasco, factors that likely reduce effective soil water availability.

The soil structural pattern combined with the greater rainfall in the carrasco may explain the delay in leaf fall in this vegetation type. Moreover, the delayed leaf flushing observed during the 2023–2024 cycle in crystalline caatinga, with leaf production occurring only in late February, further suggests a higher sensitivity of this physiognomy to interannual hydrological variability. Similar responses have been reported in other Caatinga areas, where erratic rainfall altered SOS, LOS, and ecosystem productivity (Alberton et al. 2019, 2023) even in sites with slightly deeper soils (~ 1.4 m) but reduced effective rooting depth, ~ 0.4–0.8 m (Brunello 2022).

Another factor that may contribute to the differences observed in the phenological patterns of theses physiognomies is the non-overlapping floristic composition. Carrasco exhibits higher plant species diversity, with ten species accounting for 80% of the total Importance Value Index (IVI), whereas only five species represent the same proportion in the crystalline caatinga. This greater diversity may confer higher resilience to carrasco, as it likely encompasses a broader range of phenological strategies, such as species with earlier leaf flushing or delayed leaf fall. This pattern becomes particularly evident during the driest months of the year, when some species still exhibit leaf flushing. Although both physiognomies are deciduous, carrasco as a whole does not lose all of its foliage simultaneously as the crystalline caatinga.

### Climatic factors influencing community leaf dynamics

In carrasco, the hydrothermal stress gradient was strongly associated with atmospheric variables, particularly VPD, PWD, Tmean, and water deficit, indicating that leaf activity was sensitive to evapotranspirative demand. This pattern is consistent with the local climatic conditions, characterized by more evenly distributed rainfall and deeper soils that may promote greater water retention and lower water stress (Araújo et al. 1998; Vasconcelos et al. 2010). Soil moisture and VPD are closely linked to leaf water potential (Santos et al. 2017; Paloschi et al. 2020), supporting the interpretation that, in carrasco, leaf phenology likely responds in an integrated manner to both atmospheric and edaphic controls. Higher soil moisture may therefore contribute to shaping leaf production, maintenance, and senescence.

The curves of the hydrothermal stress gradient showed negative relationships, supporting the strong association between leaf activity and atmospheric variables in carrasco, which is dominated by deciduous species. High evapotranspirative demand can induce deciduous species to reduce or interrupt their activity in order to limit transpiration and water loss (Santos et al. 2014; Paloschi et al. 2020).In addition, air temperature is closely linked to evapotranspiration and, together with water availability, may contribute to plant stress (Santos et al. 2014). Thus, in carrasco, leaf phenology also appears to be controlled by the interaction between atmospheric conditions and water availability, helping to explain the increase in Gcc under reduced hydrothermal stress. Increases in temperature enhance evaporative demand and amplify the effects of VPD on physiological processes, contributing to reduced leaf activity during warmer and drier periods (Medeiros et al. 2022). This response may also be influenced by local factors such as the occurrence of fog observed during field campaigns, given the higher elevation of carrasco sites.

Only for the carrasco, the seasonal photoperiod gradient showed a positive response, with longer day lengths associated with the initiation of leaf flushing; however, due to its relatively subtle effect on the green index, the onset of SOS remained constrained by water availability. Similar results were reported by Lima et al. (2021), who found that species with low wood density flush their leaves at the end of the dry season, prior to the onset of the first rains. Besides, species that flush leaves during the dry season in response to photoperiod cues may exhibit an adaptive strategy (Rivera et al. 2002), which could allow leaf emergence at the end of the dry season and potentially reduce herbivory pressure, since insect herbivores tend to increase following the first rainfall pulses (Aide 1992; Silva et al. 2020).

Regarding the hydroclimatic availability gradient in crystalline caatinga, although it exhibited value ranges like those of the hydrothermal stress gradient, the response curve suggested that reductions in thermal factors and evapotranspirative demand alone were insufficient to enhance leaf production, indicating a dependence on adequate soil water availability. Our results highlight how water dependence is rapidly expressed in vegetation responses. Phenocamera studies have shown that following the first precipitation pulses, leaf phenological responses can be fast, with leaf emergence occurring within a few days (Alberton et al. 2019). However, this response tends to be unstable, as increases in the green index may be quickly reduced in the absence of frequent rainfall (Alberton et al. 2019; Medeiros et al. 2022; Ramos et al. 2023). Thus, erratic rainfall and fluctuations in precipitation over time may alter patterns of leaf production and senescence in crystalline caatinga physiognomies. When precipitation is sparse or lacks temporal constancy and predictability (Zhang et al. 2025), phenological responses may not be sustained for long (Alberton et al. 2019), favoring declines in the green index and earlier leaf fall (Medeiros et al. 2022; Zhang et al. 2025), as water replenishment may be insufficient to maintain leaf production and plant water balance (Ramos et al. 2023).

## Conclusion

The woody communities of crystalline caatinga and carrasco, although both occurring within the Caatinga domain, exhibited distinct phenological strategies, reflecting different mechanisms of species adaptation to interannual hydrological and climatic variability. Our results showed that leaf phenology was primarily modulated by water availability but also displayed a strong association with thermal factors in both areas. With a more stable phenological dynamics, carrasco proved to be more predictable over time, showing lower interannual variation in SOS, EOS, and LOS, which indicates a more conservative strategy associated with atmospheric conditions and seasonal cues such as photoperiod.

In contrast, crystalline caatinga was highly sensitive to rainfall irregularity, with leaf production strongly dependent on recent precipitation pulses and rainfall continuity. Under erratic rainfall conditions or delayed rainy seasons, LOS was shortened and SOS was delayed, highlighting the greater vulnerability of crystalline caatinga to climate-driven changes in hydrological regimes. These results suggest that under scenarios of increasing climatic variability and associated events, crystalline caatinga is likely to be more sensitive, potentially leading to vegetation loss or reduction, whereas carrasco appears to be more resilient.

## Electronic Supplementary Material

Below is the link to the electronic supplementary material.

Supplementary Material 1

## Data Availability

The data supporting the findings of this study are available from the corresponding author upon reasonable request.
