# Supplementary material for: Time-lapse digital cameras reveal contrasting environmental controls of leaf phenology in different caatinga physiognomies
Source: Int J Biometeorol. 2026 Jul 13;70(7):210. doi: 10.1007/s00484-026-03261-x (PMC13364805; doi:10.1007/s00484-026-03261-x)
Supplement: Supplementary file 1 — Supplementary Material 1 [file 484_2026_3261_MOESM1_ESM.pdf]

Supplementary Information (SI)

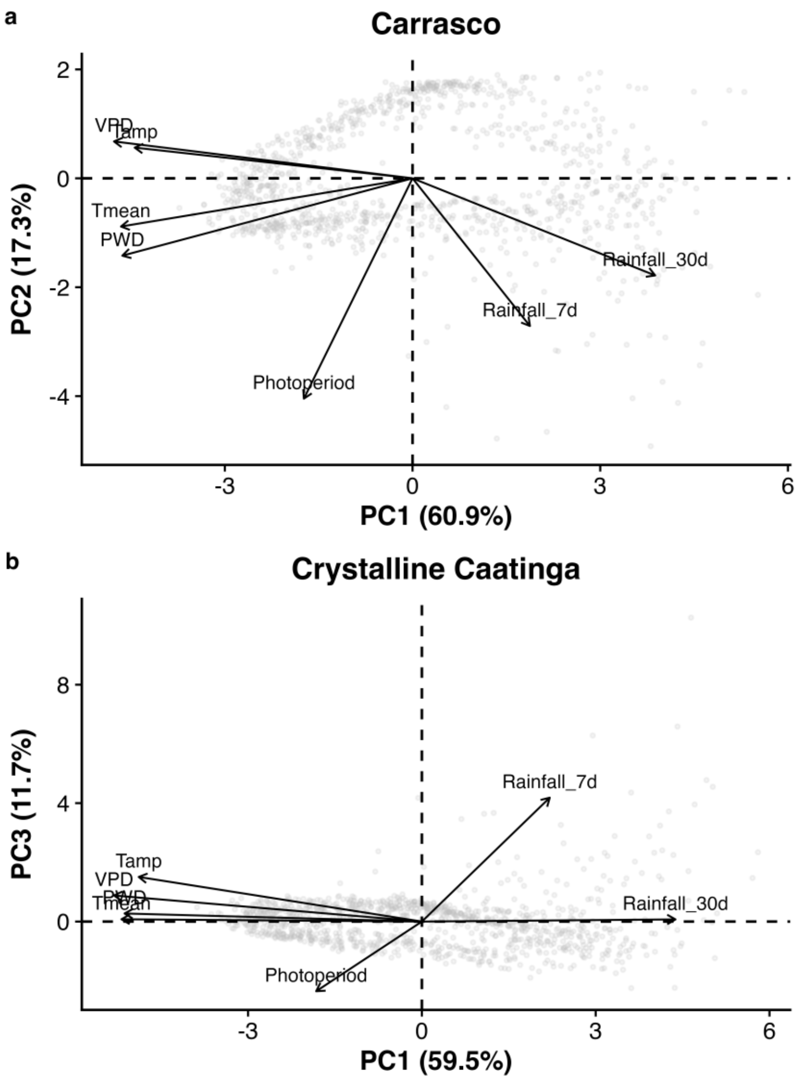

Fig. S1 PCA biplots showing climatic gradients retained in GAM analyses for (a) carrasco and (b) crystalline caatinga. Arrows represent climatic variable loadings, and gray points represent daily observations. In carrasco, PC1 represented a hydrothermal stress gradient and PC2 was associated with photoperiod seasonality. In crystalline caatinga, PC1 reflected hydroclimatic availability, whereas PC3 was primarily associated with recent rainfall pulses.

Table S1. Loadings of climatic variables on the principal components representing the main environmental gradients in carrasco and crystalline caatinga.

**Variable**	**Hydrothermal stress gradient**	**Seasonal photoperiod gradient**	**Hydroclimatic availability gradient**	**Recent rainfall pulse gradient**
**Rainfall_lag7**	0.1811	-0.4912	0.1916	0.8178
**Rainfall_cum30**	0.3744	-0.3230	0.3799	0.0142
**Tamp**	-0.4287	0.1021	-0.4242	0.2945

**Tmean**	-0.4499	-0.1604	-0.4501	0.0151
**VPD**	-0.4606	0.1227	-0.4613	0.1721
**PWD**	-0.4478	-0.2572	-0.4455	0.0534
**Photoperiod**	-0.1677	-0.7328	-0.1584	-0.4599
